# COVID-19, Companion Animals, Comparative Medicine, and One Health

**DOI:** 10.3389/fvets.2020.00522

**Published:** 2020-08-14

**Authors:** Ali Mobasheri

**Affiliations:** ^1^Research Unit of Medical Imaging, Physics and Technology, Faculty of Medicine, University of Oulu, Oulu, Finland; ^2^Department of Regenerative Medicine, State Research Institute, Centre for Innovative Medicine, Vilnius, Lithuania; ^3^University Medical Center Utrecht, Departments of Orthopedics, Rheumatology and Clinical Immunology, Utrecht, Netherlands; ^4^Centre for Sport, Exercise and Osteoarthritis Versus Arthritis, Queen's Medical Centre, Nottingham, United Kingdom

**Keywords:** COVID-19, SARS-CoV2, companion animal, zoo animal, One Health, comparative medicine, Remdesivir (RDV, GS-5734)

## Abstract

The COVID-19 pandemic in 2020 has stimulated open collaboration between different scientific and clinical disciplines like never before. Public and private partnerships continue to form in order to tackle this unprecedented global challenge. This paper highlights the importance of open collaboration and cooperation between the disciplines of medicine, veterinary medicine, and animal health sciences in the fight against COVID-19. Since the pandemic took the whole world by surprise, many existing drugs were rapidly repurposed and tested in COVID-19 clinical trials and some of the trials are revealing promising results, it is clear that the long-term solution will come in the form of vaccines. While vaccines are being developed, the antiviral agent Remdesivir (RDV, GS-5734) is being repurposed for use in human clinical trials but this is being done without acknowledging the significant efforts that went into development for treating cats with feline infectious peritonitis (FIP), a highly fatal immune-mediated vasculitis in cats which is caused by a feline coronavirus. There are many other antiviral drugs and immune modulating treatments that are currently being trialed that have animal health origins in terms of discovery and clinical development. Closer collaboration between the animal health and human health sectors is likely to accelerate progress in the fight against COVID-19. There is much that we do not yet know about COVID-19 and its causative agent SARS-CoV-2 but we will learn and progress much faster if we increase interdisciplinary collaboration and communication between human and animal health researchers and taking a genuine “One Health” approach to this and other emerging viral pathogens. Enhanced knowledge of zoonotic coronaviruses can significantly enhance our ability to fight current and future emerging coronaviruses. This article highlights the acute need for One Health and comparative medicine and the crucial importance of building on and recognizing veterinary research for addressing future human pandemics.

## Introduction

COVID-19 is a new respiratory illness in humans that affects the lungs and the airways (1). It is caused by severe acute respiratory syndrome coronavirus 2 (SARS-CoV-2) (2). In late December, 2019, an outbreak of COVID-19 was reported in Wuhan, China (3). Despite early warning about its contagious nature, SARS-CoV-2 has now spread across the globe (4), infecting more than two million people and claiming more than 243,000 lives (on 2 May 2020)^1^ On 11 March 2020, the World Health Organization (WHO), declared COVID-19 as a pandemic^2^, which is defined as the worldwide spread of new disease with major public health implications. There are some reports that the original outbreak predated December 2019 and there is emerging evidence to support this assertion.

Although COVID-19 is an emerging, rapidly evolving situation, clinical studies carried out in the last 5 months have revealed a great deal about its clinical manifestations and sequalae. The main symptoms of coronavirus (COVID-19) are fever, fatigue, continuous cough, and expectoration (sputum production) (5). The disease affects both lungs and most patients exhibit lymphopenia, increased levels of C-reactive protein (CRP), and elevated erythrocyte sedimentation rate (ESR) (5). The main clinical complications for COVID-19 patients are acute respiratory distress syndrome (ARDS) (6), which is associated with the “cytokine storm” syndrome, the uncontrolled production of pro-inflammatory mediators that contribute to ARDS (7).

The virus is highly contagious and airborne. It is spread by human-to-human transmission *via* droplets or direct contact (8). Person-to-person transmission is presumed and it is suspected to be carried by asymptomatic carriers (9). It is suspected to cause long-lasting lung damage, characterized by fibrosis. Core biopsies from post-mortem samples have revealed fibroblastic proliferation with extracellular matrix degradation and fibrin forming clusters in airspaces has been reported in (10). Other than increased CRP and elevated pro-inflammatory cytokines such as tumor necrosis factor a (TNF- a) and interleukin-6 (IL-6) (11) there are currently no biomarkers that can accurately predict clinical outcomes but some patients exhibit “dramatically” high levels of D-dimer, a by-product of blood coagulation (12, 13). Significantly higher levels of D-dimer and CRP indicate that these two proteins may be measured in combination as biomarkers of disease severity (14, 15).

In terms of treatment, there is hope for several existing antiviral agents, repurposed drugs, including a new trial that includes the use of dexamethasone^3^, and immune modulating treatments that are currently being trialed (7) and several vaccines are in development (16). Understanding immune evasion strategies of SARS-CoV2 and the resulting delayed but massive immune response and ARDS should no doubt result in the identification of disease biomarkers that predict outcomes as well as phenotype and disease stage specific treatments that will likely include both antiviral and immune modulating agents. However, it is unlikely that the above mentioned biomarkers can be used to guide treatments as the development of treatments needs to be massively accelerated to reduce the mortality rate associated with COVD-19. Another important strategy is closer co-operation between the medical, veterinary, and animal health disciplines.

This perspective article highlights the importance of taking a “One Health” approach and broader, more active and more concerted collaboration and cooperation between the disciplines of medicine, veterinary medicine, and animal health sciences in the fight against COVID-19. It is important to mention that many drugs used in human medicine were initially developed using animal models and some drugs, including Remdesivir, have been repurposed from veterinary medicine to human medicine (see later).

## One Health Approach to Studying COVID-19 and SARS CoV-2

One Health represents the collaborative efforts of multiple scientific and clinical disciplines working to attain optimal health for humans, animals, and the environment. My own unique perspective on this topic comes from my basic training in biochemistry and physiology. When a new pathogen is identified, biochemists and molecular biologist have a tendency to focus on genetic sequence and structure, and use comparative approaches to Study the new pathogen in the context of existing knowledge of similar pathogens. A good place to start is the genetic sequence of the virus and the amino acid sequence of its spike proteins, which may turn out to be important antigens and targets for development of vaccination strategies. After learning more about the sequence and the structure of the virus, return to immunology to look for ways to develop immunity to it. Let's begin with the sequence of the spike proteins. There is significant amino acid sequence homology between the spike protein epitopes of taxonomically-related coronaviruses (17). Can this knowledge help in the development of novel treatments for COVID-19? Based on the high-homology between the spike protein epitopes it has been hypothesized that past contact with infected animals may shield some humans against the circulating SARS-CoV-2 (17). This is a very interesting hypothesis that requires further attention. Since other coronaviruses can infect other animals such as cats, dogs, mice, rats, cattle, and bats, their pivotal role as “virus reservoir” needs to be considered further. Co-existence between humans and animals needs to be studied more closely because animals that have been infected with other species of coronavirus, including companion animals might act as a “beneficial” source of immune-stimulating virus particles; thus shielding against the circulating SARS CoV-2 in humans (17). However, further epidemiological and experimental studies are required to test this hypothesis. This idea is not implausible and the pioneering work that Edward Jenner did two centuries ago reminds us that taking a “One Health” approach can be extremely valuable (18). Jenner's discovery of the link between cowpox in cattle and smallpox in humans helped to lay the foundations of immunology and vaccinology, creating the first ever live vaccine: the smallpox vaccine (19).

## Canine Coronaviruses

It is important to note that canine respiratory coronaviruses are not the same as the SARS CoV-2 responsible for the COVID-19 pandemic in the human population. Dogs have had to co-evolve with their own respiratory and enteric coronaviruses. The Coronaviridae Study Group of the International Committee on Taxonomy of Viruses is the group responsible for developing the classification of viruses and taxon nomenclature of the family *Coronaviridae*. This group has recently assessed the placement of the human pathogen, tentatively named 2019-nCoV, within the Coronaviridae, providing an updated classification of the phylogeny and taxonomy of coronaviruses (2). Canine respiratory coronavirus (CRCoV) is a coronavirus of dogs, which is widespread in North America, Japan, and across Europe (20). CRCoV was detected in dogs more than 14 years ago (21). It has been associated with respiratory disease, particularly in kennel dog populations (20). The virus is highly pathogenic, causing severe lesions (22). It is genetically and antigenically distinct from enteric canine coronaviruses (23, 24), a finding which has stimulated further epidemiological research, serological surveys, and the development of new diagnostic tests. It is not clear, at this stage, if prior human exposure to CRCoV can afford any protection against later exposure to SARS CoV-2. Further research studies are required to determine if humans that co-exist with canine companions that have previously been exposed to CRCoV might develop a stronger immunity to SARS CoV-2 to those who have not had this exposure. This is purely speculative and requires further exploration.

## Can SARS-CoV-2 Infect Companion and Zoo Animals?

There has been a great deal of interest in the press about companion and zoo animals serving as reservoirs for SARS-CoV-2. It has been suggested that SARS-CoV-2 can infect cats but not dogs (25). Cats may be infected with SARS CoV-2, the coronavirus that causes COVID-19 and spread it to other cats, but according to researchers in China, dogs are not susceptible to the infection. The team at Harbin Veterinary Research Institute in China has proposed that chickens, pigs, and ducks are not likely to catch the virus. However, since COVID-19 is an emerging and rapidly evolving pandemic with the potential to use animals as reservoir hosts. There are quite a few recent reports^4^ about SARS-CoV-2 infections in mink and ferrets and linked cases of COVID-19 in humans at Dutch fur farms (26). This remind us of previous outbreaks of avian influenza virus H9N2 infections in farmed mink (27). Beyond mink and ferrets, we simply do not know much more at this stage. However, transmission from humans to dogs, domestic cats, tigers, and lions has indeed occurred. Furthermore, pigs, cats, ferrets (28), and primates have been identified as good candidates for susceptibility to SARS-CoV-2 (29). It is important to point out that SARS-CoV-2 is not originally a human virus. SARS-CoV-2 belongs to β-coronavirus family and the sequencing studies carried out so far suggest that the virus in humans is identical to the horseshoe bat coronavirus, pointing to bat as the natural, and reservoir host (16). The SARS-CoV-2 genome is closest to that of severe acute respiratory syndrome-related coronaviruses from horseshoe bats, and its receptor-binding domain is closest to that of pangolin coronaviruses (30). However, it has been proposed that the recent outbreak of COVID-19, did not come directly from pangolins (31). Recent studies also suggested that *Bovidae* and *Cricetidae* should be included in the screening of intermediate hosts for SARS-CoV-2 and could be unexplored reservoir hosts (32). The current gaps in knowledge highlight the need for field studies in the same geographical regions where the SARS-CoV-2 emerged, to look for intermediate hosts and to establish if there are any animal species that we have missed.

## The Animal Health Origin of Remdisivir

Remdesivir (RDV, GS-5734) is a broad-spectrum antiviral drug developed by the biopharmaceutical company Gilead Sciences that is currently being tested as a potential treatment for COVID-19 in international, multi-site clinical trials (33). The development of RDV was originally started by veterinary and animal professionals and focused on treating cats with feline infectious peritonitis (FIP) but this fact has been largely ignored by the press and almost forgotten by the scientific community. This is not an uncommon problem in science especially when disciplines remain focused on their own fields and do not communicate more widely. There are numerous examples of the benefits of taking a “One Health” approach in developing new therapeutics and novel medicines.

FIP is quite a rare and unusual disease in the cat caused by certain strains of the feline coronavirus. Most strains of feline coronavirus are enteric and found in the gastrointestinal tract. These enteric strains do not cause significant disease. However, in ~10% of cats infected with enteric strains of feline coronavirus, one or more mutations in the virus alter its biological behavior, resulting in leucocytes becoming infected and when this occurs, the disease is referred to as the FIP. An intense inflammatory reaction to FIP occurs around vessels in the tissues where these infected cells locate, often in the abdomen. Similar to the severe cases of SARS-CoV-2 infection in humans resulting in ARDS and over-activation of the immune system, the immune system of the cat can become over-stimulated, resulting in the development of FIP, and once a cat develops clinical FIP, the disease is usually progressive and almost always fatal without treatment, in this case Remdesivir. This is precisely why there is so much interest in the repositioning of this drug for treating SARS-CoV-2. However, Gilead Sciences is now repositioning RDV for the treatment of COVID-19 but without mentioning the significant feline coronavirus research that led to its development. The available data on the breadth and potent antiviral activity of RDV (including both contemporary human and highly divergent zoonotic coronaviruses) can significantly enhance our ability to fight current and future emerging coronaviruses (34). According to the European Medicines Agency (EMA), Remdesivir is the first biological drug against COVID-19 to be recommended for authorization in the EU. EMA's human medicines committee (CHMP) has recommended granting a conditional marketing authorization to Remdesivir for the treatment of COVID-19 in adults and adolescents from 12 years of age with pneumonia who require supplemental oxygen^5^ This is a wonderful exemplar of the need for a “One Health” approach and the importance of building on and recognizing veterinary research for addressing human pandemics (35). Drug repurposing is a reality in the pharmaceutical industry, the anthelmintic drug Ivermectin is another good example, and it can provide huge savings in valuable time and research and development budgets.

## Conclusion

Coronaviruses are a diverse group of viral pathogens. Although some of them are potentially dangerous zoonotic pathogens, many are not pathogenic. These viruses possess rapidly evolving genomes and are finding new hosts (36). In the last two decades three coronaviruses have crossed the species barrier and caused human epidemics. We have been completely unprepared for these epidemics. One of these was the recently emerged SARS-CoV-2. We were totally unprepared for the current COVID-19 pandemic. However, there were scientific papers published more than 10 years ago that predicted the emergence of such an airborne coronavirus. Nevertheless, we ignored the classic literature and we did this at our peril. A review article published in 2007, 13 years before the current COVID-19 crisis predicted this pandemic (37). The authors from the University of Hong Kong did not possess a crystal ball. Instead, they had great insight and predicted that the coronavirus would, 1 day, pose a global threat. They wrote: “The presence of a large reservoir of SARS-CoV-like viruses in horseshoe bats, together with the culture of eating exotic mammals in southern China, is a time bomb.”

Climate change and globalization are driving the destruction of natural habitats which brings humans into much closer contact with wildlife. Another example is the Nipah and Hendra virus, a virus in bats that is also transmitted the humans via intermediate hosts (38). The previous findings that horseshoe bats are the natural reservoir for SARS-CoV-like virus and that civets are the amplification host highlight the importance of wildlife and biosecurity in farms and wild animal markets. Wild animals can serve as the reservoirs and for emerging infectious diseases. There is much that we do not know about COVID-19 and the causative agent SARS-CoV-2. However, we are unlikely to progress fast unless we enhance interdisciplinary collaboration and communication (39) and take a genuine One Health approach to this and related viral pathogens. In conclusion, we will make much greater progress if we enhance collaboration and communication between human and animal health researchers, viral disease experts, wildlife ecologists, and even geographers to take a “One Health” approach to this and other emerging viral pathogens. This is not merely a battle, we are entrenched in a long-term conflict that is largely the result of human industrialization and globalization. This is a long-term conflict that cannot be won by human and animal medicine alone.

## Author Contributions

The author confirms being the sole contributor of this work and has approved it for publication.

## Conflict of Interest

The author declares that the research was conducted in the absence of any commercial or financial relationships that could be construed as a potential conflict of interest.

## Footnotes

^1^https://www.covidvisualizer.com

^2^https://www.who.int/dg/speeches/detail/who-director-general-s-opening-remarks-at-the-media-briefing-on-covid-19-11-march-2020

^3^https://www.recoverytrial.net

^4^https://www.sciencemag.org/news/2020/06/coronavirus-rips-through-dutch-mink-farms-triggering-culls-prevent-human-infections

^5^https://www.ema.europa.eu/en/news/first-covid-19-treatment-recommended-eu-authorisation
